# How Can Diet Affect the Accumulation of Advanced Glycation End-Products in the Human Body?

**DOI:** 10.3390/foods5040084

**Published:** 2016-12-06

**Authors:** Axel Guilbaud, Celine Niquet-Leridon, Eric Boulanger, Frederic J. Tessier

**Affiliations:** 1 University Lille, Inserm, CHU Lille, U995-LIRIC-Lille Inflammation Research International Center, F-59000 Lille, France; axel.guilbaud@gmail.com (A.G.); Eric.boulanger@univ-lille2.fr (E.B.); 2 UniLaSalle, EGEAL Unit, F-60026 Beauvais, France; Celine.Leridon@unilasalle.fr

**Keywords:** glycation, Maillard, advanced glycation end products, carboxymethyllysine, calorie restriction, fructose, probiotics, vitamins, diabetes, ageing

## Abstract

The accumulation of advanced glycation end products (AGEs) is associated with the complications of diabetes, kidney disease, metabolic disorders and degenerative diseases. It is recognized that the pool of glycation products found in the human body comes not only from an endogenous formation, but also from a dietary exposure to exogenous AGEs. In recent years, the development of pharmacologically-active ingredients aimed at inhibiting endogenous glycation has not been successful. Since the accumulation of AGEs in the human body appears to be progressive throughout life, an early preventive action against glycation could be effective through dietary adjustments or supplementation with purified micronutrients. The present article provides an overview of current dietary strategies tested either in vitro, in vivo or both to reduce the endogenous formation of AGEs and to limit exposure to food AGEs.

## 1. Introduction

Advanced glycation end-products (AGEs) are a complex group of molecules that have been found almost everywhere among tissues and organs of the human body [1]. Their concentration increases with age, but is also closely related to renal failure and diabetes [2]. Whether they are just a consequence of some metabolic and chemical disorders or etiologically responsible for some age-related diseases is still being debated. However, data have been accumulating for the last 20 years demonstrating more convincingly the pathophysiological role of AGEs mainly in relation to ageing and metabolic disorders.

AGEs are formed in the human body essentially by glycation [3]. This non-enzymatic reaction condenses a molecule of glucose or other reducing sugars with an amine from free amino acids or proteins, to form a Schiff base that rearranges into a ketoamine called the Amadori product. This relatively stable product can undergo further chemical reactions, such as oxidation and dehydration, to give rise to diverse AGEs. These are found on the long-lived proteins of the extracellular matrix, on short-lived plasma proteins, such as serum albumin, and on intra-cellular proteins. In all of those cases, the glycation is classified as a non-enzymatic post-translational modification of proteins. AGEs are also found as free adducts in the circulation and in the urine [4]. In this latter situation, they derive either from the proteolysis of in vivo proteins or from the absorption of dietary AGEs [5].

Helen Vlassara and her group were among the first scientists who postulated that the total load of AGEs of the human body could derive from both in vivo synthesis and dietary exposure [6]. Since then, glycation products have been classified as endogenous and exogenous AGEs, respectively. The contribution of the dietary AGEs to the total AGE load in vivo and their potentially deleterious effect on health have recently attracted considerable interest. The chemical structures of AGEs and other Maillard reaction products formed in foods during processing (mainly during heat treatment) are more heterogeneous and more complex than those formed in physiological conditions [7]. Because of this chemical diversity and other issues, estimating the contribution of dietary AGEs has been difficult. Based on a rodent study, Koschinsky et al. have estimated that 10% of the orally-ingested AGEs was absorbed, and only 30% of that was eliminated in the urine [6]. This first study revealed that a significant part of the dietary AGEs is likely to be retained in the body. Our recent studies based on exposures to dietary carboxymethyllysine (CML), one of the best studied AGEs, indicate that its chronic intake leads to an increase of its content in most organs and tissues tested [8,9,10]. Therefore, there is no doubt that exogenous AGEs contribute to the pool of AGEs found in vivo.

The term “glycotoxin” was created when the new link between the fields of the Maillard reaction in medicine (i.e., glycation) and in food science was first detected [6]. This term, which defines any reactive glycation products having a potential toxic effect in vivo, includes essentially the precursors of AGEs, among which are diverse dicarbonyl compounds. Although some reactive dicarbonyls have been found in significant amounts in foods [11], recent data revealed that it is unlikely that they can reach the circulation [12]. For instance, a study on methylglyoxal, one of the most studied intermediates, indicates that methylglyoxal coming from the diet is rapidly degraded in the gastrointestinal tract [12]. It is therefore unlikely that the exposure to food dicarbonyls will influence the methylglyoxal level in vivo or contribute to the formation of endogenous AGEs.

## 2. How Can the Presence of AGEs Be Reduced in the Human Body?

Since the discovery that the presence of AGEs in the human body could be involved in the ageing process [13], many studies have been conducted to find dietary ways to limit the accumulation of early and advanced glycation products throughout the body and eventually to slow down the potentially associated degenerative diseases.

**Figure 1:**
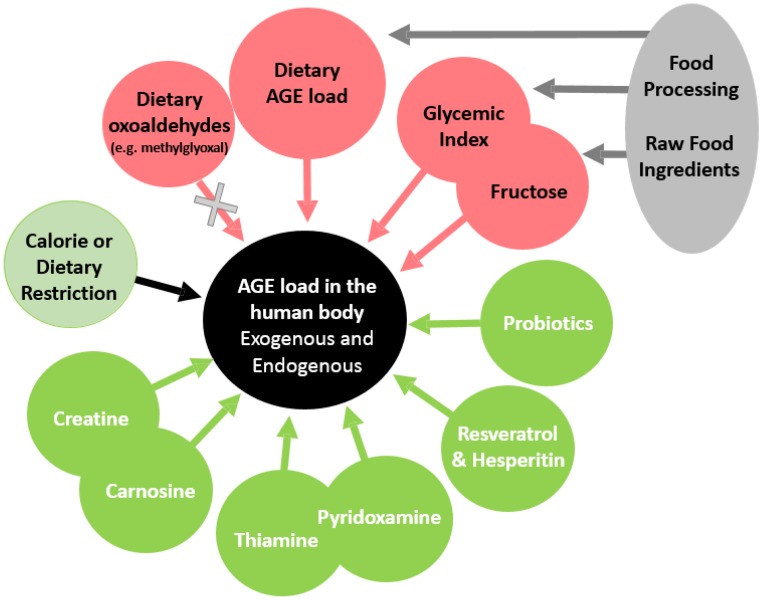
Diets and food components that affect the load of advanced glycation end-products (AGEs) in the human body.

Figure 1 shows that several constituents of foods can affect the in vivo AGE load. First of all, an overall caloric restriction was found to affect the accumulation by different amounts, some greater, some smaller, of both early and advanced glycation products. The reduction of the exposure to dietary AGEs and the reactive precursors was also found to limit the accumulation or the formation of AGEs in vivo. As a result of this, food scientists and technologists have long been exploring how potentially deleterious AGEs and other Maillard reaction products (MRPs) could be mitigated in processed foods. A more adapted heat treatment during food processing and a better selection of the raw food ingredients are known to be the two main courses of action for limiting the exposure to dietary AGEs. Special attention was given in particular to the selection of raw foods because of the influence of their carbohydrate composition on the in vivo AGE load (e.g., the amount of fructose or the proportion of complex carbohydrates in food).

Figure 1 also shows that another strategy to lessen the accumulation of AGEs was to use active ingredients derived from foods or other biological material. Numerous molecules isolated from foods (e.g., vitamins, amino acids, antioxidants) and generally tested at pharmacological doses seem to provide protection against the in vivo formation of AGEs.

The current review will not attempt to provide an exhaustive overview of the anti-AGE strategies. Instead, a couple of examples will be considered in depth.

## 3. Effects of Calorie/Dietary Restriction on the AGE Load

Caloric restriction (CR) is known to increase life span in rodents and non-human primates [14,15]. Although it would not be acceptable to apply such dieting to humans in the long term, it is used as a laboratory dietary model to study ageing mechanisms. Different CR protocols have been tested, but as far as glycation research is concerned, a 60% restriction of caloric intake was generally used. It is important to realize that the micronutrient intake was not always re-equilibrated to compensate for the restriction when the animals were calorie-restricted to 40% less that other animals given free access to food. Therefore, the term “dietary restriction” (DR) seems to be more appropriate to describe such a protocol than the term “caloric restriction”.

Studies showed that the intervention DR maintains lower blood glucose and glycated hemoglobin than ad libitum-fed rats (AL) [16,17]. These early studies led to the assumption that DR may also slow down the glycation of proteins in the body if it could reduce blood glucose and improve glucose tolerance. Some studies have been carried out to verify this hypothesis.

Rats receiving 60% of AL diet for 25 months showed an 18% to 33% reduction in the early glycation of hemoglobin, plasma proteins and skin collagen. The age-associated rate of accumulation of AGEs (CML, pentosidine and AGE-related fluorescence) was also slowed down in the skin collagen from DR rats compared to that of AL rats [18].

Other studies on rodents indicated that the glycation-lowering effect of DR differed between tissues [19] and was mainly observed at advanced ages (around 24 months) [20]. The beneficial effects of CR were not only tissue- and age-dependent, but also dependent on the strain of the animal [19]. One of the very few studies on nonhuman primates confirmed the inhibitory action of DR on the early glycation assessed by measuring furosine content in skin collagen. However, the DR did not show any effect on the advanced process assessed by CML and pentosidine quantification [21]. Evidence from this study, and others, suggests that long-term DR can inhibit early glycation (i.e., the formation of the Amadori product). This is highly dependent on the concentration of blood glucose though not advanced glycation (e.g., CML and pentosidine), which is more likely dependent on conditions other than just glycaemia, such as the lipoxidation and the overall oxidative stress. 

None of the studies presented above established a causal relationship between CR intervention and the reduction of AGEs throughout the body. At present, it remains important to understand whether the effects observed in animals were related only to an indirect better control of blood glucose or if other actions of the CR, such as a decrease in oxidative stress, were involved. Furthermore, there is no evidence that a DR lower than the ones that have been tested in animals, which would be more representative of a life-long diet suitable for humans, would significantly slow down glycation and delay associated complications in humans.

A typical DR diet is likely to be a diet that will also result in a low exposure to dietary AGEs. In addition, it is known that both DR intervention [14,15] and dietary AGE restriction intervention increase lifespan [22]. In light of these observations, it has become important to study the link between DR and AGE restrictions. For instance, Cai et al. [23] found that increasing the intake of AGEs in calorie-restricted mice erases the advantages of the CR intervention, such as a longer lifespan and lower tissue and circulation AGE levels. This observation suggests that the glycation-lowering effect of DR in the body may be partly due to a low exposure to dietary AGEs.

Studies on dietary AGEs and their impact on the in vivo AGE load are numerous. Some of the most important ones will be presented in the next paragraph.

## 4. Exposure to Dietary AGEs and Their Precursors

AGEs such as pyrraline and CML have been found at the same time in food and in vivo [24,25,26]. It was then believed, therefore, that AGEs originating from food, also called exogenous or dietary AGEs, could be partly responsible for their presence in serum and tissues. One of the first studies on this subject was conducted by He et al. [27] who fed rats with protein-bound labeled AGEs using an AGE model system with 14C and 125I. The radioactive measurement proved that the orally-absorbed AGEs, derived from an ovalbumin-glucose mixture, were distributed in several organs and tissues. Our more recent studies, based either on exposures to dietary CML or its 13C-isotopologue, indicate that the chronic intake of this AGE by mice leads to an increase of its content in the kidneys, gut, lungs, brain, heart, blood vessels and other tissues [8,9,10]. The use of a specific isotopologue of CML confirmed unequivocally the causal relationship between the CML supplied via foods and its accumulation in organs and tissues.

Apart from animal studies, some observational or interventional human studies have been conducted to confirm the contribution of exogenous AGEs to the in vivo pool of AGEs [28]. For instance, an increased serum level of AGEs was observed after the intake of a meal composed of egg white glycated with glucose [6]. Another intervention study on 64 healthy subjects confirmed that the plasma CML was slightly higher (+7%) when the volunteers ate a high MRP diet compared to a low one [29]. Although statistically significant, this increase of plasma CML was lower than expected. In addition, an observational study found an absence of correlation between dietary intake and circulating CML [30]. The low correlation or lack thereof found in those last two studies may be accounted for by the fact that the analyses were performed on fasting blood samples instead of postprandial blood samples and have, in addition, detected and quantified protein-bound CML rather than free CML. We believe, on the basis of the existing data and our own studies, that only the meals consumed within 24 h before a test will affect the level of free CML in the serum of healthy subjects.

While the influence of dietary intake of AGEs on human serum level has been proven, the same cannot be said for the relationship between dietary AGEs and tissue accumulation. Since the use of research biopsies is highly limited in clinical trials, it will be difficult to prove a causal relationship between AGE consumption and their level in the major organs and tissues. The different dietary AGE restriction studies recently published can at best only estimate the biological consequences of an oral exposure to AGEs, but not their long- or even short-term retention in the body [28,31].

Unlike the findings on AGEs derived from food, the contribution of dietary methylglyoxal and other oxoaldehydes to the neo-formation of AGEs in the body appears to be negligible. As presented in the Introduction, recent data from Degen et al. [12] revealed that methylglyoxal cannot reach the circulation, probably because of its high reactivity with amino groups in the intestinal tract.

What creates a great deal of confusion in the literature is the misuse of terms, such as “methylglyoxal-supplemented diet” [23] or “methylglyoxal^+^-diet” [32], which do not define diets supplemented directly with methylglyoxal, but diets high in BSA glycated with methylglyoxal. In this case, the terms “methylglyoxal derivatives” or “methylglyoxal-derived AGEs” would have been more appropriate.

Strategies to reduce the intake of AGEs and other MRPs have been presented in the literature pending confirmation that a dietary AGE restriction is beneficial to health. For healthy or diseased individuals who cook, it is recommended “to use mild cooking techniques instead of high-heat cooking” [33]. They may also select foods low in AGEs using available databases. There are two drawbacks here: their validity, in certain cases [34], and their accessibility to the general public, among others [35].

A better control of the Maillard reaction in products from the food industry allows desirable neoformed compounds, such as aromas and colors, to be maintained while limiting the formation of unwanted AGEs and other potentially toxic neoformed compounds [36]. Different strategies are currently being used depending on the food concerned and on the available means of improvement. These means to limit the formation of unwanted AGEs/MRPs depend as much on the selection of new raw ingredients as on the process parameters that are used [37,38].

## 5. Dietary Glycemic Index and Tissue Levels of AGEs

The glycemic index (GI) is a scale that ranks foods according to how much they raise the blood glucose level after consumption [39]. It depends not only on the quantity of carbohydrates present in a food, but also on the quality of the carbohydrates (proportion of simple and complex carbohydrates), on the overall composition of the food and on its cooking level. To put it simply, the intake of a low GI food will induce a lower increase in the blood glucose level than the intake of a high GI food. A low glycemic diet then is a diet that selects a high proportion of foods on the basis of their low GI.

Taylor and his colleagues have postulated recently that a low GI diet could limit spikes in blood glucose and consequently lower the AGE levels in vivo [40] in the same way as a long-term good glycemic control decreases AGE levels in patients with type 1 diabetes [41]. This group found that 11-month-old mice fed a high GI diet, compared to mice fed a low GI diet, had an impaired glucose tolerance. However, more importantly for our concern, these mice had at least a three-fold higher accumulation of AGEs (using protein-bound methylglyoxal-derived hydroimidazolone, MG-H1, as a marker of AGEs) in the retina, liver lens and brain [42]. The authors concluded that a high GI diet can induce what they termed a “systemic glycative stress” with pathological consequences. More work needs to be done to confirm the association between the accumulation of AGEs in the body and the intake of high-GI foods, especially with the quantification of different AGEs using accurate analytical methods. Other aspects of the carbohydrate quality should also be investigated, such as the glycemic load, the presence of dietary fibers and the proportion of sucrose, fructose and other simple carbohydrates in foods. The ability of fructose to produce more AGEs in vivo than other simple carbohydrates will be discussed in the next paragraph.

## 6. Fructose Consumption and AGE Accumulation In Vivo

The regulation of the factors that affect the Maillard reaction in food has been considered and studied for a long time. One major factor affecting the rate of the reaction is the type of carbohydrates selected in food preparation. It is well known that not all sugars have the same reactivity and lead to the formation of various MRPs in different proportions. The relationship between the type of sugars and the kinetics of the Maillard reaction has been studied in model systems to understand the formation of MRPs in foods. At least two studies found that fructose was no more reactive than glucose when browning development and sugar degradation were considered as markers of the reaction [43,44]. Another study by Kwak et al. [45], who tested the sugar reactivity on 12 amino acids, found that fructose was the least reactive among the five sugars compared.

The same research has been carried out to compare the reactivity of glucose and fructose with proteins under physiological conditions [1]. Among 12 aldoses and four ketoses tested, Bunn and Higgins found that glucose was the least reactive [46]. Compared to glucose, the authors measured a seven-fold increase in the reactivity of fructose to form Schiff bases with amino groups of hemoglobin. Another in vitro comparative study found that the fructation (i.e., glycation by fructose) of bovine serum albumin generated 10-times more fluorescence than the glycation of the same protein with glucose [47]. Since fluorescence is a marker of the advanced stage of the chemical reaction in vivo, it is was then suggested that the fructation could push forward the chemical reaction to advanced products more rapidly than glycation with glucose.

The question that we would like to address here is not only whether fructose is more reactive than glucose in physiological conditions, but more importantly, whether a high consumption of fructose is an important factor contributing to the accumulation of AGEs in vivo.

It is commonly assumed that fructose consumption has increased in the Western diet over the past five decades, mainly in the U.S., where high-fructose corn syrup (HFCS) is frequently used to replace sucrose in beverages and foods. However, Anderson explained in 2007 that the replacement of sucrose by HFCS in many products did not necessarily change the ratio of glucose and fructose consumed from sugars. Indeed, HFCS used in beverages contributes 55% percent of fructose compared with 50% of fructose from sucrose [48].

Regardless of the real situation, the belief in a high intake of fructose has gained an interest among epidemiologists and other scientists who have attempted to prove a causal correlation between dietary fructose and metabolic disorders, such as obesity, diabetes and metabolic syndrome. Some studies have confirmed a risk associated with the intake of fructose, whereas other have not reached such a conclusion [49].

The effect of a high consumption of fructose on the accumulation of AGEs in vivo was tested on rodents. The first study published in 1998 found that rats fed for one year with a normal diet and water enriched with fructose (250 g/L) had no elevated plasma glucose concentration compared to rats fed without extra fructose in the water [50]. However, the fructose-fed rats showed higher glycated hemoglobin, fructosamine and fructose levels in blood, and the collagen-linked fluorescence (a marker of AGEs) in bones was also found to be elevated in those rats compared to the control group. This was one of the first animal studies that presented evidence that a long-term intake of fructose increases the level of early glycation products in the circulation and the accumulation of AGEs in tissue. This study is nevertheless open to criticism on one particular point, which is the high dose of fructose provided in drinking water for the animals. Even so-called regular soda does not contain such a high concentration of fructose (usually <60 g/L, far below 250 g/L).

A second study aiming to understand the effects of caloric restriction also focused on the effect of different simple carbohydrates on the formation and accumulation of AGEs in vivo [51]. Tested at different ages of the animals, with or without food restriction, the source of carbohydrate was found to have almost no effect on serum glucose, glycated hemoglobin and pentosidine in collagen. It was then concluded that the total caloric intake, but not the type of carbohydrate used, had an influence on the accumulation of AGEs.

Three recent mice studies conducted by Mastrocola et al. reopened the debate about the potential toxic effect of dietary fructose. The exposure to free fructose was either via drinking water (150 g/L) [52] or animal food enriched with fructose (60% of energy coming from fructose instead of corn starch and maltodextrin for the standard food) [53,54]. Here, again, the relevance of such a high fructose dietary intake in mice to human exposure may be questioned. Bearing this limitation in mind, those studies showed a larger accumulation of CML in plasma, liver, muscle and hippocampus for the mice fed a high-fructose diet compared to mice fed a standard diet. This high CML synthesis in vivo was partly inhibited when the mice received an oral dose of pyridoxamine, an anti-glycative compound that will be described in the last paragraph of this review.

The intake of foods for which fructose is the main sugar produces a low glycemic response. For that reason, dietary fructose is often used as an alternative to other carbohydrates despite the controversy around the effect of the intake of fructose on lipemia [55]. There can be little doubt that a moderate intake of fructose significantly limits early glycation. A meta-analysis of intervention studies confirmed that the intake of fructose below 90 g per day can improve glycated hemoglobin (HbA1c) concentration in a dose-response manner [56]. With respect to the control of early glycation, a moderate substitution of fructose for sucrose or other simple sugars appears to be beneficial. A recent dietary intervention study showed that a moderate consumption of a fructose drink (60 g fructose/day) for four weeks in overweight women did not affect the urinary levels of CML and MG-H1 [57]. However, the effect of a long-term intake of fructose on the accumulation of AGEs in tissues remains to be studied on healthy volunteers.

## 7. Effects of Probiotic Supplementation on Glucose Metabolism and Glycation

The gastrointestinal tract is one of the most important interfaces between the external environment and the internal human environment. It consists of a truly integrated ecosystem named the microbial flora or microbiota. This flora consists of various microorganisms, among which are the bacteria, archaea, viruses and bacteriophages [58]. These microorganisms are known to be involved in many physiological processes, such as (1) the intake of nutrients and their metabolism; (2) the protection against any pathogen invasion; (3) the synthesis of vitamins and (4) the stimulation of the immune system [59].

Many studies have shown the importance and influence of the gut microbiota on human health [60]. A disruption of this microbiota, also called dysbiosis, can lead to physiological disorders and promote the development of some metabolic diseases, such as obesity and type 2 diabetes [61]. In recent years, studies have shown that a dysbiosis is frequently observed among people with diabetes. This is characterized by a decrease in butyrate-producing bacteria and an increase in opportunistic bacteria (*Bacteroides* and *Clostridium*) [61]. This observation led scientists to develop new antidiabetic therapies using bacterial strains, also called probiotics, such as *Lactobacillales*. Probiotics can actually induce changes in the gut microbiota, stabilize microbial communities and exert beneficial effects on the health of the host.

Studies show that the ingestion of some bacterial strains, mainly lactic acid bacteria, can reduce blood glucose levels in rodent models, such as Lepr^db/db^ mice (mice homozygous for the diabetes spontaneous mutation which is a result of a mutation in the leptin receptor gene lepr) [62], streptozotocin (STZ)-induced diabetic mice fed a high-fat diet [63], STZ-induced diabetic rats [64] and rats fed a high-fructose diet [65]. Some of these studies have also demonstrated an effect of the probiotics on the early glycation since the HbA1c content was decreased after a long-term exposure to *Lactobacillus* strains. Similar results were reported in clinical trials where volunteers consumed probiotics over a period of four to eight weeks [66,67]. These clinical studies showed a decrease in blood sugar level, oxidative stress and early glycation (HbA1c concentration). However, none of them attempted to study the potential protective effect of probiotics against the accumulation of AGEs in vivo.

## 8. Inhibition of Glycation with Isolated Products from Foods

**Figure 2:**
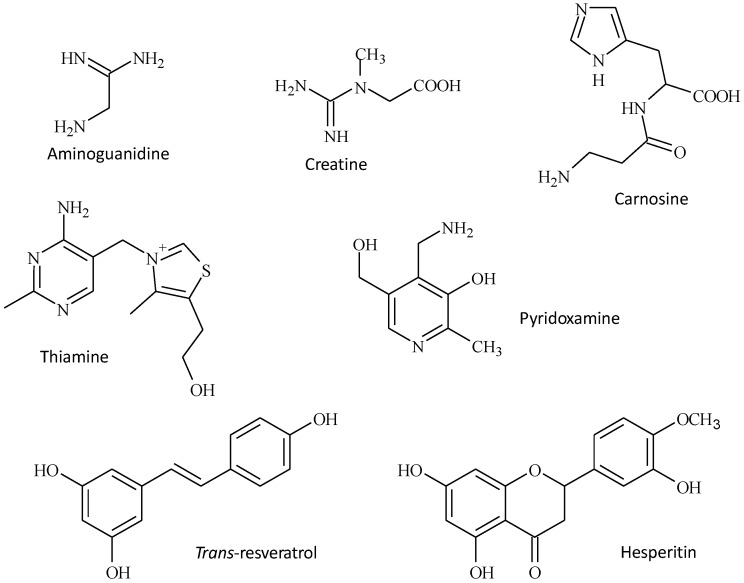
Chemical structures of aminoguanidine and natural compounds that have anti-AGE properties.

A number of AGE inhibitor compounds have been discovered in the last 20 years of which perhaps the best known is aminoguanidine (pimagedine, AG) (Figure 2). This pharmaceutical agent was the first to prove its effectiveness in inhibiting the AGE formation in vitro and in rats [68]. There are now more than 570 publications referenced in the MEDLINE database of life sciences publications when the search is based on the two keywords “glycation and AG”. This clearly demonstrates the active research around this AGE inhibitor and the high expectations created by this discovery. Unfortunately, AG has proven to be unsuitable for clinical use due to adverse side effects [69].

Some naturally-occurring compounds or their derivatives have also been tested for their potential effects against the formation of AGEs in vivo. Like AG, some of them are thought to compete with amino groups on proteins. In other words, by “sacrificing” themselves for others, they have the potential to preserve the chemical integrity of the proteins in vivo and, thus, limit the accumulation of AGEs in tissues.

Creatine is one of these compounds. It is a nitrogenous organic acid that is not only synthetized in vivo, but also derives from the diet (mainly meat and fish) (Figure 2). Just like AG, creatine is able to trap highly reactive dicarbonyl compounds and, as a consequence, may slow down the formation and the accumulation of dicarbonyl-derived AGEs [70]. The natural occurrence of creatine in vivo and the possibility of increasing its blood concentration with the consumption of animal foods or supplements are two of the positive elements that should encourage scientists to continue along this path.

Carnosine is another molecule that can act as an AGE inhibitor (Figure 2). Like most AGE inhibitors, this dipeptide seems to have more than one biological property, which can be the source of its effect on the level of AGEs in vivo. Carnosine has been described as having a hypoglycemic effect, an indirect intracellular antioxidant activity, anti-inflammatory properties and carbonyl-trapping capacities [71]. Naturally present in significant concentrations in the human lens, it was successfully tested as an antiglycation agent for the lens with the aim of delaying senile cataracts [72,73]. Retarding the tissue accumulation of AGEs with carnosine or certain derivatives of this dipeptide should be investigated further.

There are data suggesting that some B vitamins and their synthetic derivatives can reduce the formation of AGEs. Vitamin B1 and benfotiamine, its lipophilic derivative, have emerged as promising inhibitors of glycation in vivo (Figure 2). By increasing transketolase activity, both of them can increase the conversion of fructose-6-phosphate and glyceraldehyde-3-phosphate into ribose-5-phosphate [74] and therefore prevent the accumulation of triose-phosphate, which is involved in the formation of AGEs. In STZ-induced diabetic rats, the concentrations of some of the AGEs quantified in plasma and tissues decreased in the presence of high-dose thiamine [5]. However, it is not clear if the lowering effect of thiamine observed on diabetic animals could also be efficient on non-diabetic animals during the course of ageing. Clinical trials using a thiamine therapy for slowing down the accumulation of AGEs in normal subjects without apparent thiamine deficiency are now required to test the therapy fully.

Another vitamin derivative tested for its anti-glycation activity is pyridoxamine (PM) (Figure 2). This B6 vitamer [75] is found in human plasma at low concentrations (<0.03 µM) and is found in food in the form of PM-5’-phosphate. Booth et al. found that PM could prevent the transformation of Amadori products into AGEs [76,77]. Different protective effects have been attributed to PM when STZ-diabetic rats were pharmacologically supplied, and at least three mechanisms of PM’s action may explain its anti-AGE property. First of all, PM like AG can trap dicarbonyl compounds (such as methylglyoxal). It can also neutralize reactive oxygen species and inhibit the oxidative degradation of the Amadori products. Although it appears that PM can limit the formation and the accumulation of AGEs in diabetic animal models, clinical evidence of its anti-AGE effect on healthy volunteers and sick patients is still lacking [69].

A recent breakthrough is a clinical trial showing that pharmaceutical doses of two natural compounds could decrease glycation intermediates in vivo [78]. The innovative co-formulation was made of *trans*-resveratrol found in red grapes and hesperetin derived from hesperidin, which is itself found in citrus fruits (Figure 2). While inefficient when tested individually, those two compounds administrated together at high doses were found to improve metabolic health in an overweight population. They were found to decrease methylglyoxal efficiently in plasma, but surprisingly not MG-H1. This apparent lack of effect of the co-formulation on circulating AGEs may however be due to a rapid elimination of MG-H1 in the urine. A rough estimation of the urinary elimination of the endogenously-formed MG-H1 seems to indicate that the co-formulation was not only effective in reducing glycation intermediates, but also in limiting the endogenous formation of AGEs.

The efficacy of the co-formulation of *trans*-resveratrol and hesperetin was apparently due to an elimination of methylglyoxal, which resulted, indirectly, from an enhancement of the glyoxalase system (i.e., glyoxalase-1 inducer activity). Other polyphenols have been found to have a direct effect of scavenging methylglyoxal and other dicarbonyl compounds. The latter chemical property of polyphenols has been not only found at high temperature during food processing [79], but also in physiological conditions at 37 °C [80]. This preliminary observation suggests therefore that a regular intake of polyphenols from plant foods may not only sequester reactive oxygen species and the associated oxidative stress, but also trap reaction carbonyl species (i.e., dicarbonyls) and the associated glycation of proteins.

## 9. Conclusions

It is clear that preventing the formation of endogenous AGEs and the accumulation of exogenous AGEs could limit their pathophysiological effects in the human body. Besides curative pharmaceutical interventions, which are under investigation, dietary adjustments or supplementation have emerged as promising preventive therapies to slow down the accumulation of AGEs in the human body.

Decreasing the glucose uptake and controlling the blood glucose level are options that can be applied with a “slow carb” diet. Since the carbonyl stress involved in the formation of endogenous AGEs is closely linked to oxidative stress, any diet that will improve the oxidative status of humans will potentially have the additional benefit of reducing the formation of endogenous AGEs. Trapping endogenous dicarbonyl compounds, such as methylglyoxal, appears also to be a high priority in the fight against glycation. In this regard, certain natural compounds extracted from foods have been found to be good candidates, either because of their chemical affinity with the dicarbonyl compounds or their indirect action in stimulating the dicarbonyl detoxification system. While promising, the natural compounds derived from foods have been found to be effective only when extracted, purified and administrated at pharmacological dosages.
